# An Assessment of Oral-Health-Related Quality of Life and Anxiety in Early Adolescents (11–14 Years) at Their First Dental Visit: A Cross-Sectional Study

**DOI:** 10.3390/children12040428

**Published:** 2025-03-28

**Authors:** Trinidad Rincón, Cristina Gómez-Polo, Javier Montero, Daniel Curto, Adrián Curto

**Affiliations:** 1 Department of Surgery, Faculty of Medicine, University of Salamanca, Alfonso X El Sabio Avenue s/n, 37007 Salamanca, Spain; trincon@usal.es (T.R.); crisgodent@usal.es (C.G.-P.); javimont@usal.es (J.M.); 2 Department of Pathology, 12 de Octubre University Hospital, Córdoba Avenue s/n, 28041 Madrid, Spain; daniel.curto@salud.madrid.org

**Keywords:** anxiety, early adolescents, oral-health-related quality of life, pediatric dentistry

## Abstract

**Background:** Knowing the oral-health-related quality of life (OHRQoL) status of pediatric patients would be helpful in analyzing their level of dental anxiety before their first visit to a pediatric dentist. However, few studies have analyzed OHRQoL and anxiety in early adolescent patients. The aim of this study was to describe the OHRQoL and anxiety levels of early adolescents, according to age and sex, at their first dental examination. **Methods:** A cross-sectional study was conducted on early adolescents (11–14 years old) attending their first dental examination in 2023–2024. OHRQoL was assessed using the Spanish version of the Child Perceptions Questionnaire 11–14 (CPQ-Esp11-14) and anxiety using the State–Trait Anxiety Inventory in Children (STAIC). **Results:** A total of 130 early adolescents were assessed, with an average age of 12.6 years (±1.06) and an equal sex distribution (65 boys and 65 girls). Among the analyzed sample, the OHRQoL dimension with the highest score was social well-being (15.01 ± 10.7), whereas the oral symptoms dimension (8.6 ± 4.25) had the lowest impact. There were no statistically significant differences between female and male early adolescents in the anxiety state or anxiety-trait dimensions or in the dimension of OHRQoL, except for oral symptoms, which were higher in boys (9.48 ± 4.51 versus 7.72 ± 3.81). Similarly, no significant differences were found based on age regarding anxiety or OHRQoL. **Conclusions:** Considering the limitations of this study, it can be concluded that higher levels of anxiety negatively impacted the OHRQoL of the early adolescent population studied.

## 1. Introduction

Quality of life (QoL) is defined as the conditions that contribute to a person’s well-being. This concept makes it possible to analyze a person’s state of physical and mental health [1]. The concept of oral health implies the ability to speak, smile, chew, swallow, and convey emotions through facial expressions without pain or discomfort [2]. The concept of oral-health-related quality of life (OHRQoL) has been developed in connection with this term. OHRQoL has been widely applied as a measure of the impact of oral diseases and disorders on individuals and society. OHRQoL can serve as a relevant outcome measure following oral health interventions or help practitioners assess patients’ concerns [3].

The term OHRQoL has been established to assess the impact of children’s oral condition on their psychosocial well-being. Evaluating children’s self-perceived oral health through OHRQoL provides a more comprehensive assessment of their overall oral health status. Additionally, it enhances dental professionals’ understanding of their patients, promoting better cooperation from child patients during dental treatment [4,5]. In recent years, there has been a growing interest in recognizing oral health as a component of OHRQoL [4,5]. Caries is currently the main reason for consultation in the pediatric population. The severity of caries correlates positively with children’s OHRQoL and also affects their parents’ emotions [6]. OHRQoL in young patients may also be affected by factors other than oral ones, for example, systemic diseases such as congenital heart disease, coeliac disease, or renal disease [7]. Previous studies have indicated that dental treatment (e.g., exodontia, pulpectomy) improves OHRQoL in children [8,9,10].

The anxiety that children experience before and during dental treatment influences their perception of and negatively impacts their treatment [11]. Dental anxiety is related to the appearance of painful stimuli and can increase the perception of pain that patients describe, therefore hindering the cooperation of children during their treatment [12].

Children with dental fear may exhibit problematic behaviors when undergoing dental interventions. Some child patients have dental treatment anxiety and do not cooperate with their practitioner, resulting in difficulties for the practitioner and unsatisfactory treatment. These patients may even refuse treatment [13]. At a clinical level, it is difficult to distinguish between dental fear and dental anxiety [12]. Studies on dental anxiety in young adults have shown that most adults developed their fear during childhood and/or adolescence [13,14]. Recent studies even describe dental anxiety in children as a possible risk factor in certain systemic diseases [9]. The anxiety that children experience when attending a dental consultation has a negative impact on their OHRQoL and that of their families [4,15]. Implementing measures to reduce levels of dental anxiety in children can also reduce the impact of visiting the dentist on their OHRQoL and increase dental treatment cooperation and follow-up. Young patients’ dental anxiety prior to visiting the dentist is also influenced by external factors, e.g., their parents’ perception of their oral health status and previous dental experiences [1,3,5,6,7,12,13]. Among these measures to reduce dental anxiety in children are behavior management techniques, modeling techniques, and audio–visual distractions. Therefore, pediatric dentists should strive to minimize children’s stress and make it easier for them to accept dental treatment [4,16,17,18].

Early adolescence is a fundamental transition period between childhood and adulthood. In this age group, it is important to have already established correct oral hygiene guidelines and regular check-ups for children [19]. The first visit to the dentist is essential to identify whether a child is at high risk of caries and to implement preventive and interceptive tools to improve their oral health status [20,21,22]. Early dental care can reduce the need for future oral treatment and the occurrence of future dental pain or discomfort in children [23]. Not having regular check-ups at the dentist has a negative impact on the oral health status of children. As dental interventions are sometimes avoided in pediatric patients who have dental anxiety, professionals should provide a calm and comfortable environment for young patients during dental check-ups to reduce their levels of anxiety [19,24].

According to the scientific literature, no published studies have evaluated anxiety and OHRQoL in early adolescent patients during their first dental consultation.

This study’s null hypothesis stated that the anxiety experienced by early adolescent patients before their first dental examination is not associated with their OHRQoL.

The primary objective of this study was to evaluate the OHRQoL and anxiety levels reported by early adolescent patients during their first dental visit.

## 2. Materials and Methods

### 2.1. Study Design and Sample

A cross-sectional observational study was conducted on an early adolescent population of 130 participants aged 11 to 14 years. These individuals visited the University Dental Clinic of Salamanca (Salamanca, Spain) between November 2023 and November 2024. The participants were early adolescents who had not previously attended a dental consultation. According to the scientific literature [25,26,27,28], the age range of 11 to 14 years is classified as early adolescence.

This study was approved by the University’s Research Ethics Committee (protocol number 1078). All participants were informed of the methodology and objectives of the study and agreed to participate. To be included in the study, their parents or legal guardians had to sign an informed consent form. The participants were also informed that they could withdraw from the study at any time without explanation. The methodology of the study was explained verbally to the child participants in simple terms. Verbal agreement was obtained from the children. This study was planned in accordance with the STROBE (Strengthening the Reporting of Observational Studies in Epidemiology) guidelines and the Declaration of Helsinki [29].

### 2.2. Sample Size Calculation

To determine the sample size of this study, OHRQoL was considered as the main analysis variable. Within OHRQoL, we determined that the most relevant variable for patients was the oral symptoms dimension. For this variable, for a variability of ±4 points and for a maximum estimation error of ±0.75 and a 95% confidence level, the minimum sample size needed was 116 participants. By adding 10% to prevent losses, the required sample size was set to 128 subjects.

### 2.3. Eligibility Criteria for Participants

The eligibility criteria for the study included (1) early adolescents aged 11–14 years attending a dental examination for the first time; (2) patients visiting for a routine dental check-up without reporting dental pain or discomfort; and (3) patients with explicit consent from their parents and/or guardians. The exclusion criteria were (1) early adolescents with a history of dental pain; (2) patients undergoing treatment with anti-inflammatory drugs, analgesics, anxiolytics, or antidepressants; (3) individuals with physical and/or mental disabilities; (4) patients with systemic diseases; and (5) patients with craniofacial anomalies.

### 2.4. Data Collection and Variables

Data were collected from two validated questionnaires: the Child Perceptions Questionnaire validated for a population between 11 and 14 years of age (CPQ-Esp11-14), a Spanish version of the Child Perceptions Questionnaire that records OHRQoL, and the State–Trait Anxiety Inventory for Children (STAIC) [22,30].

The Child Perceptions Questionnaire 11–14 is made up of 37 items and encompasses four dimensions of OHRQoL: oral symptoms, functional limitation, emotional well-being, and social well-being. Responses were measured on a 5-point Likert scale, from 0 to 4 (0—never; 1—once or twice; 2—sometimes; 3—frequently; and 4—every day or almost every day); a higher score is indicative of worse OHRQoL [31].

The STAIC comprises two dimensions: anxiety state and anxiety-trait. The anxiety state dimension is made up of 20 items and evaluates how the subject feels (subjective perception). The anxiety-trait dimension also includes 20 items and examines anxiety as a personality trait; that is, whether the subject tends to suffer from anxiety in general [30,32].

Both questionnaires were provided to patients and were completed through interviews conducted by a single examiner with training and experience in the field of pediatric dentistry (A.C.) before the dental exploration. It was considered important that the children complete the questionnaires while the examiner was present, in isolation from their parents, to avoid parental influence on their answers. The CPQ-Esp11-14 questionnaire and the STAIC were provided in paper format to the participants.

### 2.5. Statistical Analysis

The database was created using Microsoft Office Excel (Microsoft Corporation, Redmond, WA, USA), and data analysis was performed using the Statistical Package for the Social Sciences (SPSS) version 22 (IBM corporation, Chicago, IL, USA) software package. The statistical tests included Student’s *t*-test, ANOVA, and Pearson’s correlation coefficient. A significance level of *p* < 0.05 was established.

## 3. Results

### 3.1. Baseline Characteristics of the Population

A sample of 130 early adolescents aged 11 to 14 years (mean age 12.6 ± 1.06 years) was analyzed, with an equal distribution of boys (65) and girls (65), resulting in a sex ratio of 1:1. The sample was homogeneous regarding both age and sex.

### 3.2. Oral-Health-Related Quality of Life Analysis

**Figure 1:**
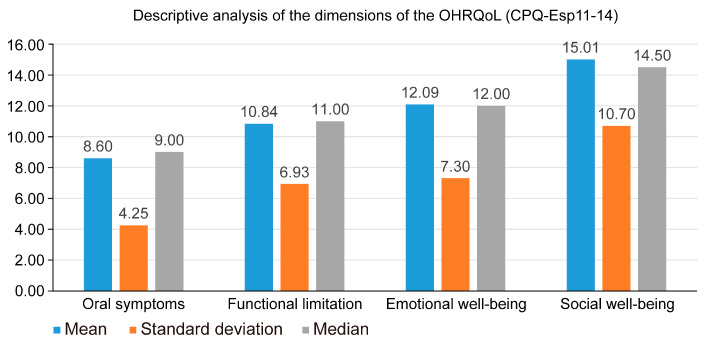
Descriptive analysis of the dimensions of the OHRQoL.

We carried out a descriptive analysis of the participants’ scores in the CPQ-Esp11-14 questionnaire in the different dimensions of OHRQoL. We observed that the dimension with the most negative impact was social well-being (15.01 ± 10.7), while the oral symptoms dimension had the lowest score (8.6 ± 4.25). It should be noted that the social well-being dimension had an average score almost double that of the oral symptoms dimension (Table 1) (Figure 1).

The following is a description of the range of scores obtained in the different dimensions of oral quality of life in the CPQ-Esp_11–14_. In the oral symptoms dimension, the scores ranged between 0 and 22 (possible range: 0–24); in the functional limitation dimension, the scores ranged between 0 and 28 (possible range: 0–36); in the emotional well-being dimension, the scores ranged between 0 and 31 (possible range: 0–36); and in the social well-being dimension, the scores ranged between 0 and 42 (possible range: 0–52).

The influence of the patients’ sex and age on their OHRQoL was evaluated (Table 2 and Table 3). In relation to sex, it was observed that there were only statistically significant differences in the oral symptoms dimension; in this dimension, boys obtained a higher score (9.48 ± 4.51), which led to a worse perception of dental treatment, associated with greater discomfort in the oral cavity, compared to girls (7.72 ± 3.81) (*p* = 0.018). In the other dimensions of the CPQ-Esp11–14 questionnaire, we observed that boys obtained higher scores than girls, but there were no significant differences. The influence of age on OHRQoL was analyzed by grouping the participants into four age ranges (11, 12, 13, and 14 years), and we observed that age did not influence the OHRQoL described by the patients, although a trend was reported in which younger participants obtained higher scores.

### 3.3. Anxiety Analysis

**Figure 2:**
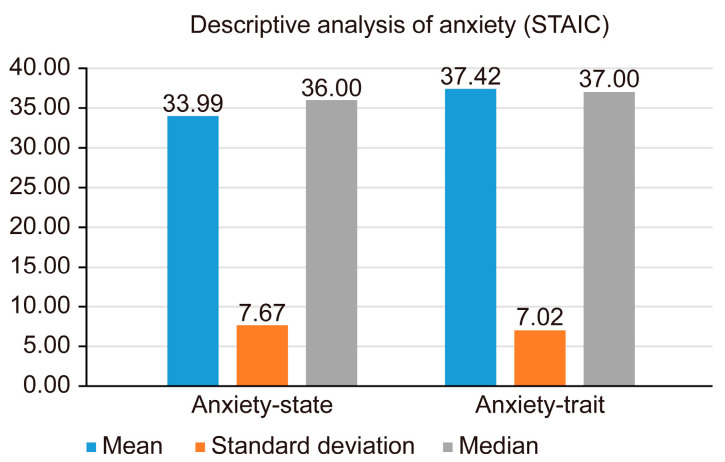
Descriptive analysis of anxiety.

The participants’ anxiety levels were assessed during their first dental examination. Upon analyzing the anxiety state and anxiety-trait scores using the STAIC, it was found that the average anxiety-trait score (37.42 ± 7.02; median = 37.0) was slightly higher than the average anxiety-state score (33.99 ± 7.67; median = 36.0) (Table 4) (Figure 2).

In addition to analyzing the influence of sex and age on OHRQoL, the impact of these factors on the patients’ anxiety levels was also assessed. Table 5 and Table 6 show the results of the analysis of the average anxiety-state and anxiety-trait scores based on sex and age. No statistically significant differences were observed between anxiety state and anxiety-trait based on sex or age.

### 3.4. Correlation Between Oral-Health-Related Quality of Life and Anxiety

Finally, anxiety-state and anxiety-trait from the STAIC were correlated with the four dimensions of the OHRQoL of the CPQ-Esp11–14 questionnaire. We found that there were highly significant correlations (*p* < 0.01) between anxiety-state and anxiety-trait and the different dimensions of OHRQoL, whereby higher values of anxiety in patients at their first dental consultation were associated with worse OHRQoL, especially in the dimensions of functional limitation, emotional well-being, and social well-being (Table 7).

## 4. Discussion

It is important to highlight the significance of considering the anxiety of child patients attending a dental practice for the first time, as well as their oral quality of life. It is likely that after dental treatment, patients’ oral quality of life will improve. The aim of the present study was to analyze the anxiety levels and OHRQoL of early adolescent patients before their first dental consultation. A statistically significant correlation was observed between anxiety and OHRQoL. Based on the results reported here, the proposed null hypothesis was rejected as a directly proportional relationship between anxiety levels and the impact of anxiety on the oral quality of life of patients before their first dental examination was observed.

Previous studies have analyzed the relationship between OHRQoL and anxiety in adult populations and concluded that anxiety in adults has a negative influence on OHRQoL [33,34,35,36]; however, the questionnaires used in these studies are not validated for the early adolescent population.

A study conducted by Samami M found that almost 60% of the adult participants evaluated had dental anxiety according to the Modified Dental Anxiety Scale (MDAs) [33].

To the best of our knowledge, there are no published studies evaluating anxiety and OHRQoL in early adolescent patients at their first dental consultation. Similar studies were previously carried out by Vanhée T [36] and Asokan S [37], who analyzed the influence of molar incisive hypomineralization on dental anxiety and OHRQoL in patients aged between 8 and 10 years old. Molar incisor hypomineralization had no significant impact on dental anxiety or OHRQoL in the population analyzed. Asokan S [37] analyzed the correlation between IQ, dental anxiety, and OHRQoL in children between 10 and 11 years of age. The author concluded that children with higher IQs experienced a smaller impact on their OHRQoL. However, the authors did not evaluate patients who had not previously attended a dental consultation.

In the present study, data collection was based on questionnaires validated and widely used in previous studies [38,39,40,41,42]. The Spanish version of the Child Perceptions Questionnaire (CPQ-Esp11–14) was used to analyze OHRQoL in children, and the State–Trait Anxiety Questionnaire (STAIC) was used to evaluate anxiety. The STAIC has previously been used to assess anxiety in children within dental settings, but it is not a commonly used method for analyzing dental anxiety. Therefore, it was deemed suitable for use in this study to evaluate its effectiveness in the dental field [17,43,44,45,46,47,48], as we wanted to use a questionnaire to analyze general anxiety, not a specific questionnaire for dental anxiety.

This study found that the dimension of the CPQ-Esp11–14 with the highest score was that of social well-being (15.01 ± 10.7), while the oral symptoms dimension had the lowest score (8.6 ± 4.25). The oral symptoms dimension analyzes whether patients have pain, gingival bleeding, wounds in the mouth, etc.; therefore, these results indicate that the participants did not experience a significant effect on their oral health status; on the contrary, they did indicate that their oral health status negatively affected their social interactions. Based on these results, it is worth mentioning that there was a wide range of responses from the lowest possible scores to medium scores, but no scores were recorded at the highest possible levels; therefore, it can be concluded that, at a general level, the oral quality of life of the patients analyzed was good prior to their first dental consultation. Contrasting results have been described by other authors, such as Pohl MB [46] and Piovensan C [47], who reported that the oral symptoms dimension had the highest score. These results may have been obtained because the studies were conducted in a population in a developing country.

Regarding anxiety, a higher score was observed for anxiety traits than for anxiety states. These results were similar to those reported by other authors [17,49,50], although it should be noted that these studies did not determine the level of anxiety at participants’ first dental consultations. Panagiotou E [48] found no correlation between caries or gingival/periodontal disease and dental anxiety levels in children.

When analyzing the relationship between anxiety and OHRQoL in this study, a directly proportional correlation was observed: higher levels of anxiety in patients had a negative impact on their OHRQoL; the highest correlations were observed between functional limitation and anxiety-state (r = 0.50) and between emotional well-being and anxiety-trait (r = 0.54).

In the present study, when comparing OHRQoL based on sex, we observed that there were only significant differences in the oral symptoms dimension of CPQ-Esp11–14. In this dimension, boys obtained higher OHRQoL scores compared with girls. No significant influence was observed between the sexes in the other dimensions. Previous studies report that sex does not influence OHRQoL, as described, for example, by Asokan S [37], Vanhée T [36], and ÇarıkçıoğluB [50]. Other authors describe better OHRQoL in boys compared with girls [35,39,40,41,42,51,52,53,54]. This association could be explained by girls often worrying more about their health problems, both functional and esthetic. In contrast, Thiruvenkadam G [55] and Kumar S [56] found that boys had worse OHRQoL; it should be noted that these two authors used questionnaires other than the CPQ11-14 to analyze OHRoL.

In the present study, age did not influence OHRQoL (*p* > 0.05), a conclusion also reached by other authors [34,36,45,48]. It should be noted that in the present study, an age range of 11 to 14 years was chosen for analysis; it would be interesting to expand participants’ age range and evaluate the influence of age on OHRQoL.

In this study, anxiety levels were not influenced by either the age or sex of the participants. Other authors, such as Vanhée T, also found no relationship between age and anxiety levels in children [36]. Regarding sex, some studies have described higher levels of anxiety in girls compared with boys [36,57]; however, these studies analyzed the influence of incisor–molar hypomineralization on anxiety levels.

As indicated above, the social well-being dimension had the highest impact on oral quality of life (15.01 ± 10.7); in contrast, the oral symptoms dimension had the lowest score (8.6 ± 4.25). In the oral symptoms dimension, the scores ranged from 0 to 22, covering almost the entire expected range. No patients obtained high scores in the remaining dimensions of the CPQ-Esp11–14 (functional limitation, emotional well-being, and social well-being); therefore, it can be concluded that the OHRQoL of the patients studied was not poor before their first dental visit. It is noteworthy that the oral symptoms dimension had the smallest impact on the patients despite the analysis of a population of early adolescents who had not seen a dentist before.

This study has some limitations. The primary limitation is that the study included patients within a narrow age range (11 to 14 years old). Another limitation is that the evaluated population was taken from a single university care center, which may have resulted in sampling bias; for example, patients attending this center may have had fewer economic resources for maintaining their oral health. Another limitation of this study was the time taken by the participants to complete the two questionnaires; the authors wanted to use the original versions of both tools and not shorter versions of the questionnaires. The results described in this study should be considered in the context of the population studied.

In future research, it would be beneficial to expand the age range of the sample population, as well as to design multicenter and longitudinal studies to assess the maturity of the scores. Future research could also analyze the influence of different sociodemographic, economic, and clinical data of child patients (for example, parents’ educational level, parents’ perception of their children’s oral health status, parents’ previous dental experiences, brushing frequency, caries indices, periodontal health status) on anxiety and OHRQoL.

This study has several strengths. First, the sample analyzed is balanced in terms of sex and age range. Additionally, this study is one of the first published works where anxiety and OHRQoL are evaluated in early adolescent patients who have never previously attended a dental consultation. In future studies, it would be useful to analyze the effects of different anxiety management techniques in early adolescent and adolescent patients. The implementation of objective measures to analyze OHRQoL and anxiety before a dental intervention in early adolescents may help identify specific fears, which could facilitate dental professionals’ work, patient collaboration, and treatment follow-up.

## 5. Conclusions

In this study, anxiety was associated with the OHRQoL of early adolescent patients attending their first dental consultation. OHRQoL and anxiety were not influenced by patient age or sex, with the exception of the oral symptoms dimension, where boys experienced a more negative effect than girls.

## Figures and Tables

**Table 1:** Descriptive analysis of the dimensions of oral-health-related quality of life (CPQ-Esp11–14 questionnaire).

Dimensions of CPQ-Esp_11–14_	Mean	Standard Deviation	Median
**Oral symptoms**	8.6	4.25	9.0
**Functional limitation**	10.84	6.93	11.0
**Emotional well-being**	12.09	7.3	12.0
**Social well-being**	15.01	10.7	14.5

**Table 2:** Comparison of the dimensions of oral-health-related quality of life (CPQ-Esp11–14 questionnaire) based on sex.

Dimensions of CPQ-Esp_11–14_	Mean (±SD)		Student’s *t*-Test	*p*-Value
	Boys (n = 65)	Girls (n = 65)		
**Oral symptoms**	9.48 (±4.51)	7.72 (±3.81)	2.4	0.018 *
**Functional limitation**	11.2 (±6.76)	10.48 (±7.13)	0.59	0.554
**Emotional well-being**	12.46 (±7.24)	11.72 (±7.39)	0.58	0.566
**Social well-being**	15.09 (±10.45)	14.92 (±11.02)	0.09	0.929

* = significant (*p* < 0.05).

**Table 3:** Comparison of the dimensions of oral-health-related quality of life (CPQ-Esp11–14 questionnaire) based on age.

Dimensions of CPQ-Esp_11–14_	Mean (±SD)				Anova	*p*-Value
	11 Years (n = 28)	12 Years (n = 32)	13 Years (n = 41)	14 Years (n = 29)		
**Oral symptoms**	10.11 (±4.12)	8.66 (±4.34)	8.32 (±4.68)	7.48 (±3.32)	1.95	0.125
**Functional limitation**	12.0 (±6.92)	10.09 (±6.7)	10.73 (±7.41)	10.69 (±6.7)	0.39	0.762
**Emotional well-being**	12.75 (±7.79)	11.59 (±6.85)	12.27 (±7.52)	11.76 (±7.29)	0.15	0.929
**Social well-being**	16.79 (±10.55)	13.81 (±10.0)	15.27 (±11.27)	14.24 (±11.06)	0.44	0.723

**Table 4:** Descriptive analysis of anxiety using State–Trait Anxiety Inventory in Children (STAIC).

STAIC Dimensions	Mean	Standard Deviation	Median
**Anxiety-state**	33.99	7.67	36.0
**Anxiety-trait**	37.42	7.02	37.0

**Table 5:** Comparison of the dimensions of anxiety using the State–Trait Anxiety Inventory in Children (STAIC) based on sex.

STAIC Dimensions	Mean (±SD)		Student’s *t*-Test	*p*-Value
	Boys (n = 65)	Girls (n = 65)		
**Anxiety-state**	33.95 (±7.64)	34.03 (±7.76)	0.05	0.955
**Anxiety-trait**	37.58 (±6.64)	37.26 (±7.42)	0.26	0.794

**Table 6:** Comparison of the dimensions of anxiety using the State–Trait Anxiety Inventory in Children (STAIC) based on age.

STAIC Dimensions	Mean (±SD)				Anova	*p*-Value
	11 Years (n = 28)	12 Years (n = 32)	13 Years (n = 41)	14 Years (n = 29)		
**Anxiety-state**	35.71 (±6.99)	33.13 (±7.73)	33.34 (±7.58)	34.21 (±8.46)	0.71	0.549
**Anxiety-trait**	38.11 (±6.47)	36.81 (±6.78)	37.8 (±6.61)	36.9 (±8.48)	0.26	0.854

**Table 7:** Association between the dimensions of oral-health-related quality of life (CPQ-Esp11–14) and the dimensions of anxiety (State–Trait Anxiety Inventory in Children).

	Anxiety-State		Anxiety-Trait	
	Correlation Coefficient	*p*-Value	Correlation Coefficient	*p*-Value
**Oral symptoms**	0.37	<0.01 **	0.40	<0.01 **
**Functional limitation**	0.50	<0.01 **	0.43	<0.01 **
**Emotional well-being**	0.48	<0.01 **	0.54	<0.01 **
**Social well-being**	0.48	<0.01 **	0.47	<0.01 **

** = highly significant (*p* < 0.01).

## Data Availability

The data presented in this study are available upon request from the corresponding author due to ethical reasons.

## Abbreviations

The following abbreviations are used in this manuscript:

OHRQoL	Oral-health-related quality of life
STAIC	State–Trait Anxiety Inventory in Children
STROBE	Strengthening the Reporting of Observational Studies in Epidemiology
MDAs	Modified Dental Anxiety Scale
CPQ-Esp11-14	Spanish version of the Child Perceptions Questionnaire 11–14
